# The Sphingolipid-Modulating Drug Opaganib Protects against Radiation-Induced Lung Inflammation and Fibrosis: Potential Uses as a Medical Countermeasure and in Cancer Radiotherapy

**DOI:** 10.3390/ijms25042322

**Published:** 2024-02-15

**Authors:** Lynn W. Maines, Staci N. Keller, Ryan A. Smith, Cecelia L. Green, Charles D. Smith

**Affiliations:** Apogee Biotechnology Corporation, 1214 Research Blvd, Suite 2015, Hummelstown, PA 17036, USA

**Keywords:** opaganib, ABC294640, sphingolipid, sphingosine kinase, fibrosis, radioprotection, medical countermeasure

## Abstract

Fibrosis is a chronic pathology resulting from excessive deposition of extracellular matrix components that leads to the loss of tissue function. Pulmonary fibrosis can follow a variety of diverse insults including ischemia, respiratory infection, or exposure to ionizing radiation. Consequently, treatments that attenuate the development of debilitating fibrosis are in desperate need across a range of conditions. Sphingolipid metabolism is a critical regulator of cell proliferation, apoptosis, autophagy, and pathologic inflammation, processes that are all involved in fibrosis. Opaganib (formerly ABC294640) is the first-in-class investigational drug targeting sphingolipid metabolism for the treatment of cancer and inflammatory diseases. Opaganib inhibits key enzymes in sphingolipid metabolism, including sphingosine kinase-2 and dihydroceramide desaturase, thereby reducing inflammation and promoting autophagy. Herein, we demonstrate in mouse models of lung damage following exposure to ionizing radiation that opaganib significantly improved long-term survival associated with reduced lung fibrosis, suppression of granulocyte infiltration, and reduced expression of IL-6 and TNFα at 180 days after radiation. These data further demonstrate that sphingolipid metabolism is a critical regulator of fibrogenesis, and specifically show that opaganib suppresses radiation-induced pulmonary inflammation and fibrosis. Because opaganib has demonstrated an excellent safety profile during clinical testing in other diseases (cancer and COVID-19), the present studies support additional clinical trials with this drug in patients at risk for pulmonary fibrosis.

## 1. Introduction

Fibrosis is a dysregulated repair process that progressively replaces normal tissue with fibrotic tissue leading to impaired organ function, and thereby playing a role in up to 45% of all deaths in the industrialized world [1]. In particular, pulmonary fibrosis can occur from exposure to toxins such as asbestos, coal dust, silica, cigarette smoke, oxidative drugs or radiation, as well as from autoimmune diseases or viral infections, including COVID-19. Regardless of the initial insult or pathological condition, fibrosis is driven by the chronic activity of inflammatory cytokines, particularly TGFβ, that promote cellular fibrogenic processes [2] and aberrant activation of granulocytes and fibroblasts [3,4]. Exposure to ionizing radiation (IR) is used for approximately 50% of patients receiving cancer therapy. Furthermore, multiple government agencies are supporting research to identify drugs, i.e., medical countermeasures (MCMs), that can mitigate morbidity and mortality from unintended exposure to IR from accidental or terroristic nuclear incidents. Inflammatory responses to IR-induced tissue damage can lead to morbidity from multiple organ impairments, including pulmonary fibrosis. Consequently, there is high interest in developing fibrosis inhibitors for multiple indications, and effort has focused on taming the inflammatory cells and cytokines that drive fibrosis.

Sphingolipid metabolism is the subject of broad investigation (reviewed in [5,6,7]), including evaluation of its roles in lung diseases such as pneumonitis and pulmonary fibrosis [2,8,9,10,11,12]. The involvement of sphingolipids in radiation-induced lung injury has recently been reviewed [13]. Ceramide is produced by the hydrolysis of sphingomyelin in response to several growth stimulatory (e.g., growth factors and oncoproteins) and inflammatory (e.g., cytokines and radiation) signals. Ceramide is hydrolyzed by ceramidases to produce sphingosine, which is phosphorylated by sphingosine kinase-1 (SK1) and sphingosine kinase-2 (SK2) to produce sphingosine 1-phosphate (S1P). Because sphingolipids mediate the pathologies of a variety of inflammatory diseases, many studies have addressed the possibility of suppressing S1P formation as an innovative approach to therapy (reviewed in [5,6,7]). In particular, the actions of inflammatory cytokines are dependent on S1P production by SKs, making these kinases plausible targets for new anti-inflammatory drugs [6,14,15]. Of particular relevance to fibrosis, interactions between sphingolipid metabolism and responses to TFG-β have also been widely discussed [16,17,18,19,20,21,22,23]. Overall, there is substantial evidence supporting the hypothesis that SK inhibitors have excellent potential for suppressing acute inflammation as well as chronic inflammatory and fibrotic pathologies.

Opaganib (3-(4-chlorophenyl)-N-(pyridin-4-ylmethyl)-1-adamantanecarboxamide, hydrochloride salt; also known as ABC294640) is an orally active, isozyme-selective inhibitor of SK2, and is competitive with respect to sphingosine [24,25]. Opaganib decreases S1P production and elevates dihydroceramides [26], thereby suppresses signaling through pERK, pAKT, and NFκB, and promoting autophagy [5,24,25]. Opaganib has antitumor activity in a wide range of mouse models (reviewed in [5]), and has shown promise in oncology [27,28] and COVID-19 [15,29] clinical trials. Additionally, opaganib has in vivo anti-inflammatory activity in several rodent models, including: ulcerative colitis, Crohn’s disease, colitis-driven colon cancer, vascular permeability, rheumatoid arthritis, osteoarthritis, liver transplantation, hepatic ischemia-reperfusion injury, and acute kidney injury (Reviewed in [15], as well as bacterial pneumonia [30], lupus nephritis [31], psoriasis [32], renal fibrosis [33], and gastrointestinal acute radiation syndrome [34]. In models of inflammatory bowel disease, opaganib decreased levels of TNFα, IL-1β, IFN-γ, IL-6, and S1P levels in colon tissue [35]. In liver IR injury, opaganib reduced TLR4 expression, NFκB activation, TNFα, IL-1β, and CXCL-10 production, adhesion molecule expression, and liver infiltration of granulocytes [36]. An essential role of SK2 in *Pseudomonas aeruginosa* (*PA*)-induced lung inflammation has been described [37,38], and the ability of opaganib to nearly completely ameliorate *PA*-induced lung injury in mice with suppression of inflammatory cell accumulation and IL-6, TNFα, and H_2_O_2_ was demonstrated [30]. Thus, opaganib has excellent potential to attenuate pulmonary inflammatory conditions.

We have previously demonstrated that opaganib suppresses gastrointestinal acute radiation syndrome (GI-ARS) in mice after total body irradiation (TBI) with partial body shielding [34]. In the present study, we investigated the therapeutic activity of opaganib in models of lung inflammation induced by exposure to ionizing radiation.

## 2. Results

**Figure 1:**
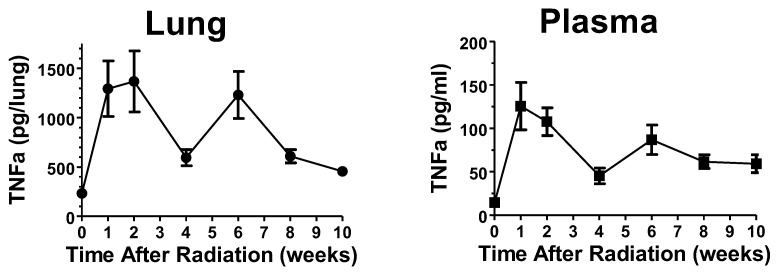
Effects of 14 Gy thoracic radiation on TNFα levels. Mice were exposed to 14 Gy thoracic radiation and then euthanized at the indicated times. TNFα levels in lung homogenates and plasma were determined by ELISA.

Time course of pulmonary inflammation following thoracic irradiation. We first determined the time course of pulmonary inflammation in a thoracic irradiation model. Mice were anesthetized and exposed to 14 Gy thoracic radiation using a Varian Clinac linear accelerator, as detailed in the Materials and Methods section. Groups of 8 mice were euthanized immediately or at 1, 2, 4, 6, 8, or 10 weeks after radiation, and samples of plasma and lungs were assayed for TNFα (56 mice total). As demonstrated in Figure 1, levels of TNFα in the lung and the plasma samples were elevated in a biphasic manner, consistent with an initial rapid inflammatory response and a secondary inflammatory response peaking at Week 6. This experiment suggests that treatment with opaganib may be optimal when given prior to, or as soon as practical after, irradiation, and/or in Weeks 4–8 post-irradiation.

**Figure 2:**
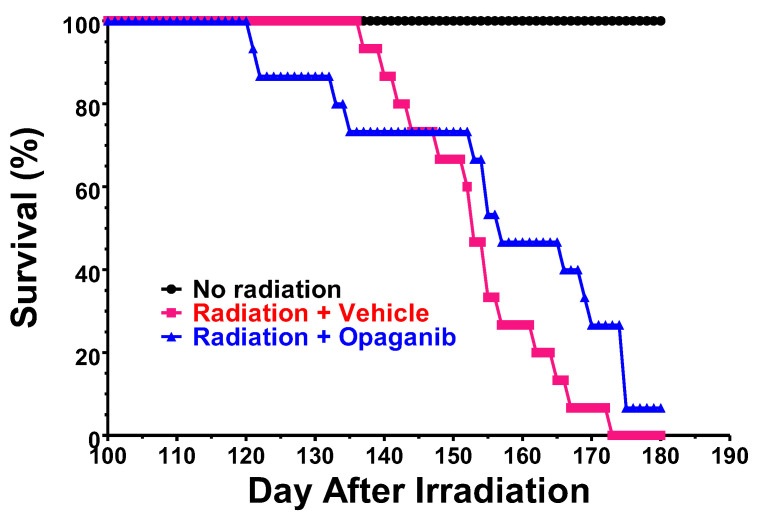
Effect of acute administration of opaganib following partially shielded radiation on survival. Male C57BL/6 mice were exposed to 0 (Group A) or 16 Gy TBI with 5% bone marrow shielding and treated with 0 (Group B) or 150 mg/kg opaganib (Group C) twice daily for 5 days. On Day 30, 15 mice/Group were selected, monitored daily and euthanized according to the IACUC-approved protocol.

Efficacy of early treatment with opaganib. We previously reported that administration of opaganib to mice exposed to high-dose TBI with 5% bone marrow shielding protected the animals against GI-ARS and markedly improved 30-day survival [34]. In those experiments, opaganib treatment was not started until 24 h after radiation exposure to model use of the drug as a medical countermeasure (MCM) to mitigate GI-ARS from unintentional, e.g., accidental or terroristic, radiation exposure. We therefore conducted a similar short-term, post-radiation exposure experiment to determine if the opaganib treatment regimen that was active against GI-ARS improved the longer-term survival of mice. Therefore, male C57BL/6 mice were exposed to 16 Gy TBI with 5% bone marrow shielding and then treated with 0 or 150 mg/kg opaganib twice-daily (BID) for 5 days, beginning 24 h after irradiation (Groups B and C, respectively). Group A mice were not irradiated or drug-treated as a control. On Day 30, i.e. after some animals had died from GI-ARS, surviving mice (N = 15/group) were randomly selected and monitored to Day 180. The survival curves for these Groups for Days 100–180 are shown in Figure 2 (N = 15/group, 45 total)). No deaths occurred in non-irradiated (Group A) mice in this period. However, 100% of vehicle-treated mice irradiated at 16.0 Gy (Group B) died between Days 137 and 173. The early opaganib-treated mice (Group C) experienced a modest survival benefit compared with Group B. Specifically, Kaplan–Meier estimates of the median survival and associated 95% confidence intervals (Cis) indicate increased median survival (127 days for Group C compared with 123 days for Group B; *p* = 0.069). Furthermore, a Cox Proportional Hazards regression model indicates that there is a 45% reduction in the risk of death in Group C (Hazard Ratio = 0.55, 95% CI = 0.26 to 1.15; *p* = 0.11). While these differences do not quite reach a significance level of 0.05, they support our hypothesis that blunting the radiation-induced inflammatory response with opaganib may provide protection against delayed pathologies.

Optimization of the opaganib-treatment schedule. Because the inflammatory response to radiation is biphasic and prolonged for at least 8 weeks, dosing with opaganib through a broader timeframe may be superior to the acute schedule used above. We conducted multiple experiments with different treatment schedules to address this issue.

**Experiment 1.** In the first experiment, mice (15 total) were treated orally with opaganib either prior to radiation exposure (as would be the case for cancer patients receiving radiotherapy), after radiation through the initial inflammatory period (as would be the case for use of a MCM by victims of unintended exposure), or both before and after radiation (Table 1). The radiation dose was reduced to 15 Gy to allow longer survival, and mice were monitored for toxicity and euthanized at 180 days after irradiation in the thoracic irradiation model. A non-irradiated group of mice was also monitored for 180 days as a control.

**Figure 3:**
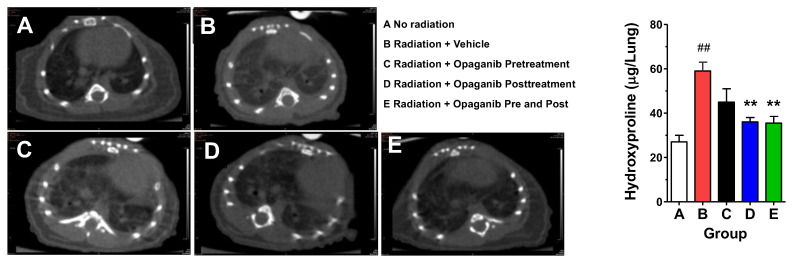
Effect of opaganib on lung density and collagen deposition following thoracic radiation. **Left Panel**. Mice were exposed to radiation and opaganib, as indicated in Table 1. On Day 180 after radiation exposure, the mouse thoraxes were imaged using a Siemens Inveon Micro-CT/PET. Representative sections from mice in each treatment group are shown. **Right Panel**. On Day 180 after radiation exposure, the lungs were harvested and the amounts of hydroxyproline were quantified. ## *p* < 0.01 versus Group A; ** *p* < 0.01 versus Group B.

Immediately prior to euthanization on Day 180, the extent of lung damage was assessed using micro-CT imaging (Figure 3). The lung scans of mice receiving radiation but no drug treatment (Group B) were more opaque than control mice (Group A), reflecting the higher density of the fibrotic lung tissue in these animals. Pretreatment alone with opaganib (Group C) did not have an observable effect on lung density; however, treatment with multiple doses of opaganib between Days 14 and 24 (Groups D and E) substantially reduced the degree of lung damage assessed by imaging. Collagen levels in the lungs were estimated by measuring the amount of hydroxyproline in HCl-digested lung tissue. As shown in Figure 3, radiation exposure (Group B) markedly increased (*p* < 0.01) the amounts of hydroxyproline in the lungs at Day 180, compared with controls (Group A). Treatment with opaganib (Groups D and E) strongly reduced the levels of hydroxyproline in the lungs (*p* < 0.01) compared with radiation alone. In all, the data indicate that a single dose of opaganib prior to radiation provides some protection against the development of pulmonary fibrosis. However, the repeated use of opaganib during the initial inflammatory period after irradiation appears to provide the greatest reduction in the fibrotic cascade.

**Experiment 2.** Because Experiment 1 demonstrated that treatment with opaganib through the initial post-radiation inflammatory period suppresses long-term lung damage, the effects of post-radiation treatment with opaganib on lung inflammation at 6 weeks were assessed. As indicated in Table 2, opaganib was given as a single dose; short-term for 5 days; or long-term for 6 weeks in the thoracic irradiation model. A non-irradiated group of mice was also monitored for 180 days as a control (40 total mice).

**Figure 4:**
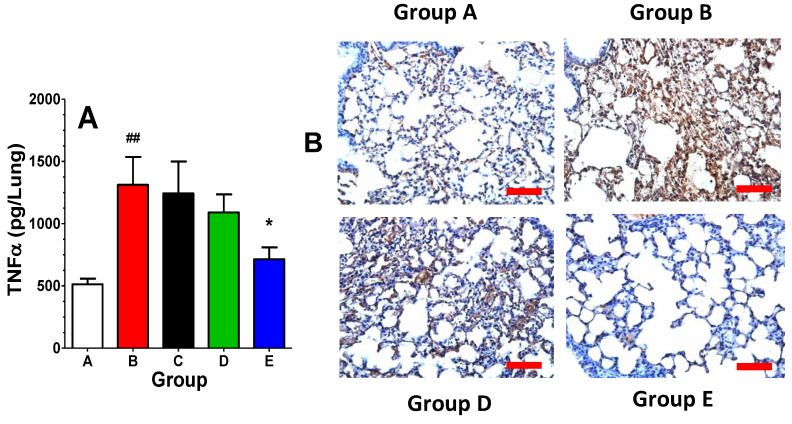
Effect of opaganib on radiation-induced TNFα accumulation and macrophage infiltration in the lungs. (**A**) Mice were exposed to radiation and opaganib, as indicated in Table 2. At 6 weeks after radiation, mice were euthanized and lungs were harvested, washed, and TNFα levels were measured using ELISA. ^##^ *p* < 0.01 versus A, * *p* < 0.05 versus B. (**B**) At 6 weeks after radiation, mice were euthanized and lungs were harvested, formalin-fixed, paraffin-embedded, and sliced. Representative sections were stained for macrophages using anti-CD68 antibodies. Red bar indicates 150 µm.

As indicated in Figure 4A, lung TNFα levels were markedly elevated in the radiation alone group (Group B) compared with control mice (Group A) at 6 weeks after irradiation. A single dose of opaganib given 24 h after irradiation (Group C) did not suppress the inflammatory response at 6 weeks, consistent with the marginal benefit from single-dose opaganib in Experiment 1. Repeated daily dosing for the first 5 days after irradiation provided only a modest reduction in lung TNFα at 6 weeks. However, prolonged treatment with opaganib throughout the first inflammatory period (Group E) almost completely suppressed the elevation of lung TNFα in response to irradiation. Formalin-fixed, paraffin-embedded sections from the lungs harvested at 6 weeks post-irradiation were stained with anti-CD68 antibodies to visualize the extent of macrophage infiltration into the lungs. Compared with control lungs (Group A), lungs from radiation alone mice (Group B) contained numerous immunoreactive macrophages (Figure 4B). Macrophage infiltration was reduced in Group D, and was essentially eliminated in the long-term opaganib-treated animals (Group E). In all, the data indicate that a single dose of opaganib following radiation is insufficient to protect against lung inflammation. However, the repeated use of opaganib after irradiation provides substantial protection against subsequent lung inflammation.

**Experiment 3.** An additional long-term study was conducted to further compare the efficacy of opaganib as a MCM when given only in the initial inflammatory phase, only in the delayed inflammatory phase, or in both phases. Mice were exposed to TBI with 5% bone marrow shielding, treated with vehicle or opaganib beginning 24 h later, and monitored for 180 days for survival. Mice were then sacrificed for analyses of inflammatory and fibrotic endpoints. As shown in Table 3, groups of mice were irradiated at 15.25 Gy and then treated with vehicle or opaganib (100 mg/kg): twice daily for 3 days beginning at 24 h after radiation; twice daily for 3 days beginning at 24 h after radiation and then once daily on Days 31–45; or once daily on Days 31–45 after radiation. On Day 30, i.e. after some animals had died from GI-ARS, surviving mice (N = 10/group) were randomly selected and monitored to Day 180. A non-irradiated group of mice (N = 5) was also monitored for 180 days as a control (45 total mice).

**Figure 5:**
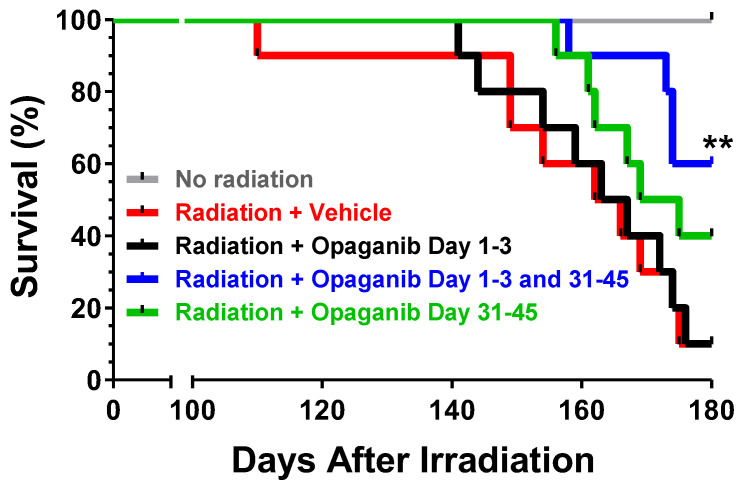
Effect of opaganib schedule following partially shielded radiation on survival. Male C57BL/6 mice were exposed to 0 (Group A) or 15.25 Gy radiation, and treated with: vehicle at all dosing times (Group B); 100 mg/kg opaganib twice-daily, beginning 24 h after irradiation, for 3 days only (Group C); 100 mg/kg opaganib twice-daily, beginning 24 h after irradiation for 3 days, and then again once daily with 100 mg/kg opaganib on Day 31-45 (Group D), or 100 mg/kg opaganib once daily on Day 31-45 only (Group E). Mice were monitored daily and euthanized according to the IACUC-approved protocol. ** *p* < 0.01 vs. Radiation + Vehicle (Group B).

The Kaplan–Meier survival curves for the treatment groups for Days 100–180 are shown in Figure 5. No deaths occurred in non-irradiated mice (Group A) in this period. However, 90% of vehicle-treated mice irradiated at 15.25 Gy (Group B) died between Days 110 and 175. The Day 1–3-only treatment group (Group C) also experienced 90% mortality between Days 141 and 176. Mice in the Day 31–45-only treatment group (Group E) experienced a 40% survival rate (4-fold of control) that is near statistical significance compared with the radiation + vehicle group (*p* = 0.08). Most impressively, the Day 1–3 plus 31–45 treatment group (Group D) demonstrated a highly statistically significant (*p* = 0.008) improvement in survival at Day 180 (60% compared with 10% for controls). Thus, treating with opaganib during both phases of inflammation provided the greatest improvement in survival.

**Figure 6:**
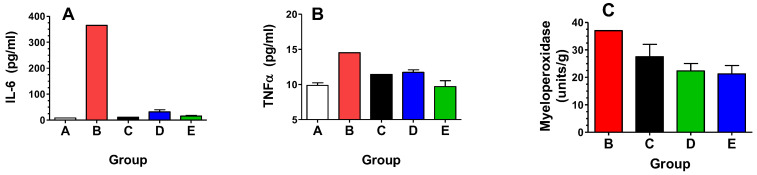
Effects of opaganib on inflammatory markers in lungs at 180 days post irradiation. Lungs from mice described in Table 3 were evaluated for inflammatory cytokine levels (**A**,**B**) or myeloperoxidase activity (**C**).

Surviving mice were euthanized on Day 180 for secondary endpoint analyses, yielding sample sizes of 5, 1, 1, 6, and 4 for Groups A, B, C, D, and E, respectively. After euthanasia, the left lung was fixed in formalin for histology, and the right lung was snap frozen for biochemical analyses. Unfixed lung samples were homogenized in PBS and centrifuged, and the supernatants were analyzed for expression of the key inflammatory cytokines IL-6 and TNFα. As shown in Figure 6A,B, levels of IL-6 and TNFα were elevated in the radiation + Vehicle group (Group B), as compared to unirradiated controls (Group A), indicating a prolonged inflammatory state in the lungs. All the opaganib treatment groups (Groups C, D and E) had markedly lower expression levels of IL-6 than did the radiation-alone group; however, a statistical comparison was not possible because of the small sample number. Lung levels of TNFα were only slightly elevated on Day 180, and were essentially equal to unirradiated mice in all the opaganib treatment groups. As a measure of granulocyte infiltration, myeloperoxidase (MPO) activity was also measured in the lungs of mice on Day 180. As shown in Figure 6C, MPO activity was also reduced in opaganib-treated mice compared with the irradiated + Vehicle group (Group B).

**Figure 7:**
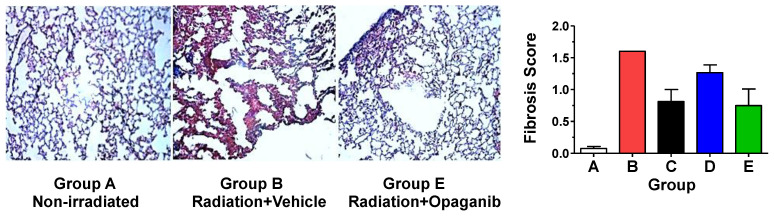
Effect of opaganib on radiation-induced pulmonary fibrosis. **Left Panels**: Representative sections of mouse lungs stained for collagen from animals described in Table 3 at 180 days post-irradiation. **Right Panel**: The fibrosis score according to the Ashcroft scale was determined at 180 days post-irradiation.

The formalin-fixed, paraffin-embedded lungs were sectioned and stained with Masson’s trichrome to visualize collagen deposition (Figure 7). Fibrosis was absent in unirradiated animals (Group A), present in the irradiated + Vehicle group (Group B), and reduced in opaganib-treated mice, as represented by a section from a mouse treated with opaganib on days 31–45 (Group E). Staining patterns were similarly reduced in tissue from mice treated on Day 1–3 and 31–45 (Group D). Additionally, the Fibrosis Score in the lung sections was determined in a blinded fashion by an external veterinary pathologist using the Ashcroft scale, scoring 10 non-overlapping 10x fields per mouse. As shown in Figure 7, there was very little fibrosis in sections from the unirradiated group (Group A). The irradiated + Vehicle group (Group B) had the highest fibrosis score, although this reached only a mild degree of fibrosis because mice were euthanized on Day 180. Nonetheless, treatment with opaganib in either the early inflammatory phase (Group C) or the delayed inflammatory phase (Group E) substantially reduced the average fibrosis score in the lungs of irradiated mice.

## 3. Discussion

It is well established that sphingolipid metabolism regulates inflammation underlying several disease states, and, in particular, many studies have shown that SK activity regulates pathologic inflammation [39,40,41]. Sphingosine 1-phosphate induces NFκB [42], which in turn can increase levels of the proinflammatory enzymes nitric oxide synthase and cyclooxygenase-2 [43,44,45], generating prostaglandins and oxidative and nitrative stress that exacerbates inflammation [46]. Inflammatory cytokines induce adhesion molecule expression via activation of SK and NFκB, and these proteins facilitate infiltration of neutrophils and macrophages into inflamed tissue. Sphingosine 1-phosphate also promotes granulocyte activation, leading to production of reactive oxygen species [47,48], and activates S1P receptors, further promoting inflammatory cascades at the site of tissue damage [49,50,51]. In parallel, a growing body of knowledge demonstrates the roles of sphingolipids in exacerbating many of the processes that lead to fibrosis [2]. For example, sphingolipids mediate the effects of TGFβ1 on processes that are critical to fibrosis, such as the expression of fibronectin [33]. Overall, a strong body of evidence demonstrates that activation of SKs alters sphingolipid metabolism in favor of S1P formation, resulting in pro-inflammatory and pro-fibrotic responses.

Because radiation-induced pulmonary fibrosis is mediated by excessive inflammation regulated by sphingolipids [16,17,18,19,20,21,22,23], we hypothesized that opaganib may decrease pulmonary damage from ionizing radiation. In previously reported work, we demonstrated that oral treatment of mice with opaganib 2 h before exposure to TBI, or IR with partial bone marrow shielding, provides substantial protection against GI-ARS associated with suppression of radiation-induced elevations of S1P and TNFα in the small intestines [34]. In the partially bone marrow shielded model, opaganib improved survival when administered 4 h before or 24 h after radiation exposure, and was particularly effective when given both prior to and following radiation [34]. In several mouse tumor models, the combination of opaganib with radiation provided the optimal suppression of tumor growth without increasing toxicity to the animals [34]. Overall, opaganib substantially protected normal tissue from radiation damage that may occur through unintended exposure or cancer radiotherapy.

We next focused attention on assessing the efficacy of opaganib against delayed effects of radiation exposure, which could occur from induce either intended (cancer radiotherapy) or unintended (nuclear accident or terrorist attack) exposure to IR. Even having drugs and MCMs that mitigate hematologic and gastrointestinal damage from IR, it remains necessary to identify drugs that mitigate radiation-induced longer-term pathologies such as fibrosis. Work by others lent support for studying the effects of opaganib on fibrosis. In particular, Zhu et al. demonstrated that opaganib inhibits TGFβ1-induced expression of fibronectin and α-smooth muscle actin in renal fibroblasts, as well as TGFβ1-induced activation of STAT3^Y705^ and AKT/GSK3α/β signaling [33]. Moreover, this paper demonstrated marked protection by opaganib in kidneys of mice following the unilateral ureteral obstruction (UUO) fibrosis model. Fibronectin and α smooth muscle actin levels were sharply reduced in opaganib-treated mouse kidneys, compared with saline-treated controls. On a tissue level, tubular atrophy and collagen deposition were markedly alleviated in opaganib-treated mice [33].

The current studies indicate that opaganib attenuates pulmonary inflammation and injury following exposure to IR in two independent in vivo models. Temporal complexity in the systemic inflammatory response to radiation was demonstrated as a biphasic production of TNFα consisting of an acute phase for the 2 weeks following radiation and a delayed phase at 4–6 weeks following radiation. Interestingly, a biphasic inflammatory response was also documented in human patients receiving lung radiation for lung cancer [52,53] supporting the clinical relevance of the current mouse models.

In most studies described herein, treatment with opaganib was withheld until 24 h after radiation exposure to mimic the scenario for treatment as a MCM, i.e., for unintended radiation exposure (accidental, terrorist, or military), in which there would be a delay before treatment. The current results demonstrate that administration opaganib starting at +24 h following radiation can provide a significant survival advantage against pulmonary inflammation-associated delayed death. The greatest improvement in survival was observed when opaganib was given in both the early treatment period (Day 1–3) and the delayed treatment period (Day 31–45). It is interesting to note that protection of the mice from GI-ARS by opaganib (mortality at Day 30 in the Days 1–3-only treatment group was reduced by 50% compared with vehicle-treated irradiated mice) does not provide statistical protection against delayed mortality. However, administration of opaganib on Day 31–45 only did provide a substantial survival benefit, and treatment during both phases of the inflammation response (Day 1–3 and Day 31–45) following radiation exposure was even more successful for suppression of long-term lung damage. Therefore, the delayed inflammatory response may be more important for driving long-term, fibrotic pathologies than is the early inflammatory stage. Of course, the exposed subject must survive the GI-ARS period to experience long-term survival, and so the optimal treatment paradigm appears to involve a split-schedule of administration of opaganib, in which it is provided at Days 1–3 to protect against GI-ARS and then again during the secondary inflammatory period to protect against pulmonary fibrosis. It would be informative to test an additional treatment group in which opaganib is administered daily from radiation to euthanasia to determine if a continuous treatment protocol is superior to the split schedule.

It should be noted that the lung Fibrosis Score and levels of collagen at Day 180 were relatively modest, even though the dose of radiation was sufficient to kill nearly all the vehicle-treated mice. Analyses of the effects of opaganib on survival and pulmonary fibrosis at 180 Days were the IACUC protocol-defined endpoints of the current studies. It would be informative to carry mice for longer periods after radiation at lower doses to allow greater changes in the mechanistic secondary endpoints, and the current data provides rationale and a foundation for larger and longer future studies.

It is useful to note that cancer patients receiving radiotherapy do so under planned exposure protocols, which allows for the pretreatment of these patients prior to exposure to IR. In the GI-ARS model, opaganib had excellent preventative activity when given prior to radiation exposures [34]. Therefore, we expect that administration of opaganib prior to radiation exposure will be substantially more protective against pulmonary inflammation and fibrosis than when the drug is given only after radiation. Therefore, the efficacy of prophylactic treatments with opaganib on fibrosis hold excellent promise for improving the quality of life in cancer patients undergoing radiotherapy.

Overall, opaganib is a promising drug suited for dual tract development as an MCM and as a cancer therapeutic. We believe that the ability of opaganib to provide therapeutic benefit against both GI-ARS and delayed pulmonary damage is an important aspect for using this drug as an MCM. Specifically, the schedule of opaganib administration can be optimized to improve long-term survival using a single drug rather than requiring treatment with multiple agents with potential pharmacokinetic, metabolic, and/or toxicity interactions. Additionally, opaganib has high chemical stability and is easily administered as a gelatin capsule, making it very suitable for stockpiling and use as an MCM following a mass casualty event. Further development of opaganib as an MCM for mitigating GI-ARS and delayed radiation toxicities, such as pulmonary fibrosis, will require confirmation of improved survival in radiation-exposed nonhuman primates.

In parallel, opaganib is a viable candidate for clinical trials in patients at risk for pulmonary inflammation and fibrosis. Because of the chronic nature of fibrosis, a useful drug must not only be effective, but also safe for a prolonged dosing. Importantly, the clinical safety profile of opaganib given twice-daily to cancer patients for up to 18 months supports extended continuous dosing regimens. Additionally, opaganib has also shown excellent safety and preliminary efficacy in patients with compromised lung function. Specifically, a Phase 2/3 multinational randomized, placebo-controlled study demonstrated the safety of opaganib in patients hospitalized with severe COVID-19, and a clinical benefit to patients requiring oxygen supplementation of 60% or less (62% reduction in rate of ventilation and death) [54]. To date, more than 470 people have been treated with opaganib in oncology and COVID-19 clinical trials, demonstrating the excellent safety profile of the drug even in severely compromised patients. Finally, because signaling pathways promoting fibrosis are generally similar regardless of insult, tissue or organ, opaganib may have efficacy for additional fibrogenic conditions.

## 4. Materials and Methods

### 4.1. Materials

Opaganib (GMP-grade) was synthesized according to French et al. [24]. For drug treatments, opaganib was suspended in 0.375% Tween-80 in PBS and administered by oral gavage. Hydroxyproline assay kits were purchased from Sigma-Aldrich (St. Louis, MO, USA). Anti-CD68 antibodies were from Abcam (Waltham, MA, USA).

### 4.2. Thoracic Irradiation Model

In all in vivo experiments, opaganib was dissolved in 0.375% Tween-80 and administered by oral gavage. Control mice received oral gavage of the solvent alone. C57BL/6 mice (8–12-week-old females) were exposed to ionizing radiation using a Varian Clinac 600C/D linear accelerator producing 6 MV photons. The collimator field size was set to 2 × 35 cm. Mice were positioned side-by-side so that the short axis of the field covered the lung area of each mouse only, minimizing radiation dose to the non-targeted areas. Solid water with a thickness of 5 cm was placed under the mice during delivery to provide backscatter radiation. The dose was prescribed to a depth of 0.5 cm. Prior to any animal irradiation, dosimetry was verified using EBT2 Gafchromic (Ashland Inc., Covington, KY, USA) film and solid water. Film was positioned under 0.5 cm of solid water and exposed for a set number of monitor units, using the same beam energy, field size, and source-to-surface distance to be used during animal irradiation. The films were analyzed, and it was determined that, at the prescription depth of 0.5 cm, the dose rate conversion was 0.876 cGy/MU. In addition, it was determined that, at the prescription depth, a dose uniformity of ±5% could be achieved over the central 12 cm of the field. This 12 cm constraint was used when determining the number of mice that could be irradiated at one time. Finally, film was irradiated at a depth of 1 cm to determine the approximate posterior exit dose experienced by the mice. At a depth of 1 cm, the average dose increased by approximately 13% compared to the dose at 0.5 cm. This increase in dose was expected, as both 0.5 cm and 1 cm were within the buildup region for 6 MV photons. Each experiment included a non-irradiated control group so that the normal values of the experimental endpoints could be compared. All animals were observed for toxicity immediately after radiation exposure and daily until the end of the study. Mice were euthanized according to the IACUC-approved protocol.

### 4.3. Total Body Irradiation with 5% Bone Marrow Shielding Model

All animals were housed and cared for under the standard operating procedures of CiToxLAB North America, (Laval, QC, Canada) operating under the Canadian Council on Animal Care guidelines. Groups of male C57BL/6 (10 weeks of age; Jackson Labs, Bar Harbor, ME, USA) received a TBI dose of 16.0 or 15.25 Gy, with 5% partial bone marrow shielding achieved with a hind pelvic leg being extended and shielded by a carrobend structure. The radiation dose was quantified using a Farmer ionization chamber connected to an electrometer. The dose rate of the ^60^Co gamma source was fixed at approximately 60 cGy per minute. Following radiation, supportive care of acidified water and wetted chow on the bottom of the cages in dishes was provided to all mice. For drug treatments, opaganib was suspended in 0.375% Tween-80 in PBS with constant stirring of the drug solution, and administered by oral gavage. Mice were monitored daily, and euthanized according to the IACUC-approved protocol. Surviving mice were euthanized on Day 180 for secondary endpoint analyses. Upon euthanization, both sides of lungs (with bronchi) of up to five animals per group were retained.

### 4.4. MicroCT Imaging

At 180 days, mice (3 from each of the indicated treatment groups) were imaged using a Siemens Inveon Micro-CT/PET (Siemens Medical Solutions, Knoxville, TN, USA). Transmission images were acquired for each animal at 80 kV and 500 μA and a spatial resolution of ∼30 μm using Inveon Research Workplace software Version 1.5 (Siemens, Berlin, Germany).

### 4.5. Endpoint Analyses

At the time of euthanization, both lungs were removed and rinsed with PBS. The right lobes were snap frozen in liquid nitrogen and stored at −70 °C until assay. The lung samples were thawed and homogenized in 10 volumes (*w*/*v*) of cold PBS, and the homogenate was centrifuged at 22,000× *g* for 15 min to prepare supernatants for analysis. The left lobes were infused with neutral buffered 10% formalin, and stored at room temperature in neutral buffered 10% formalin until embedding in paraffin and thin sectioning according to standard protocols.

A: The following analyses were conducted on lysates from the right lungs:

Cytokine levels: Supernatants from the homogenates of each lung were used to determine the levels of TNFα or IL-6 using Luminex assays performed by the Cytokine Core Laboratory at the University of Maryland, Baltimore.

Collagen quantification: Hydroxyproline levels were determined as a quantitative measure of collagen accumulation in the lung using assay kits (Sigma) and normalized to tissue wet weight.

Leukocyte infiltration: Myeloperoxidase (MPO) activity was determined as a measure of granulocyte and monocyte infiltration into the lung. Supernatants of the lung homogenates were assayed for MPO by quantifying the metabolism of tetramethylbenzidine, as described by Fitzpatrick et al. [55], and normalized to tissue wet weight.

B: The following analyses were conducted on the thin sections from fixed lungs:

Collagen deposition: Staining with Masson’s trichrome was used to visualize collagen deposition in the lung thin sections. The extent of fibrosis was assessed using the Ashcroft scale, which grades fibrosis on a 0–8 scale with: 0 being normal; 1 being minimal fibrous thickening of alveolar or bronchiolar walls; 2–3 being moderate thickening without definite damage to lung structure and small fibrous bands or masses; 4–6 being increased fibrosis with definite damage to the lung structure and fibrous masses present; and 7–8 being severe distortion of structure and large fibrous areas/total fibrous obliteration of the field [56].

Macrophage infiltration: Thin sections were stained with anti-CD68 antibodies to assess macrophage infiltration, according to standard protocols.

### 4.6. Statistics

Mouse survival rates were compared using the Kaplan–Meier approach with the Gehan-Breslow-Wilcoxon test using GraphPad Prism 5 software (Version 5.00). Other data were analyzed by one-way ANOVA using the Tukey post hoc test. Differences were considered to be statistically significant when *p* < 0.05. Error bars in Figures represent the mean ± standard deviation of the treatment groups calculated with GraphPad Prism.

## Figures and Tables

**Table 1:** Treatment parameters for Experiment 1. Female C57BL/6 mice were treated with the indicated thoracic radiation and opaganib doses, as indicated in the treatment schedule (N = 3/group).

Group	Radiation (Gy)	Opaganib (mg/kg)	Treatment Schedule
A	0	0	Vehicle at 2 h before radiation
B	15	0	Vehicle at 2 h before radiation
C	15	100	Single dose at 2 h before radiation
D	15	100	Daily doses on Days 14–18 and 21, 22, and 24
E	15	100	Single dose at 2 h before radiation, followed by daily doses on Day 14–18 and 21, 22, and 24

**Table 2:** Treatment parameters for Experiment 2. Female C57BL/6 mice were treated with the indicated thoracic radiation and opaganib doses, as indicated in the treatment schedule (N = 8/group).

Group	Radiation (Gy)	Opaganib (mg/kg)	Treatment Schedule
A	0	0	Vehicle at 24 h after radiation
B	14	0	Vehicle at 24 h after radiation
C	14	100	Single dose at 24 h after radiation
D	14	100	Once daily for 5 days, beginning 24 h after radiation
E	14	100	Three times per week for 6 weeks, beginning 24 h after radiation

**Table 3:** Treatment parameters for Experiment 3. Male C57BL/6 mice were treated with the indicated radiation and opaganib doses, as indicated in the treatment schedule (N = 10/group).

Group	Radiation (Gy)	Opaganib (mg/kg)	Treatment Schedule
A	0	0	Vehicle
B	15.25	0	Vehicle
C	15.25	100	Two times daily for 3 days, beginning at 24 h after radiation
D	15.25	100	Two times daily for 3 days, beginning at 24 h after radiation, and then once daily on Day 31–45.
E	15.25	100	Once daily on Day 31–45 after radiation

## Data Availability

All data is included in the article.
